# Recent advances in asthma genetics

**DOI:** 10.1186/1465-9921-9-4

**Published:** 2008-01-15

**Authors:** Jian Zhang, Peter D Paré, Andrew J Sandford

**Affiliations:** 1 James Hogg iCAPTURE Center for Cardiovascular and Pulmonary Research, St. Paul's Hospital, Vancouver, B.C., V6Z 1Y6, Canada

## Abstract

There are over 100 genes that have been reported to be associated with asthma or related phenotypes. In 2006–2007 alone there were 53 novel candidate gene associations reported in the literature. Replication of genetic associations and demonstration of a functional mechanism for the associated variants are needed to confirm an asthma susceptibility gene. For most of the candidate genes there is little functional information. In a previous review by Hoffjan *et al*. published in 2003, functional information was reported for 40 polymorphisms and here we list another 22 genes which have such data. Some important genes such as filaggrin, interleukin-13, interleukin-17 and the cysteinyl leukotriene receptor-1 which not only were replicated by independent association studies but also have functional data are reviewed in this article.

## Background

Asthma is a disease of chronic airway inflammation that affects nearly 155 million individuals worldwide [1,2]. Like other atopic diseases, asthma is a complex disorder caused by interactions between multiple genes of small to modest effect and equally important environmental factors. Asthma has an important genetic component but no clear pattern of inheritance, and heritability estimates of asthma vary between 36–79% [3-5].

It is possible to define a categorical phenotype for studies of asthma genetics. However, the heterogeneity of the disease makes this problematic. Therefore, many studies have used quantitative phenotypes as intermediates e.g. skin test responses and total or specific serum IgE. In this case, "affected" individuals can be defined as those exceeding a certain threshold at the extreme of the distribution. Alternatively, an approach called the quantitative trait locus (QTL) method allows the entire distribution to be used in the analysis. Quantitative traits are most likely due to the influence of several alleles at multiple loci interacting to cause the phenotype. In a more recent development, gene expression array technology has been employed to use gene expression as an outcome variable. For example, a genome-wide association of gene expression was performed by Dixon *et al. *using a cohort of families with an asthmatic proband [6]. In this study, genes involved in response to unfolded protein, regulation of progression though the cell cycle, RNA processing, DNA repair, immune responses and apoptosis showed highly heritable traits. In addition, the global map made by the study will be a excellent resource for selecting candidate genes [6].

Efforts to identify asthma genes have been carried out in various laboratories around the world and because of the complexity and heterogeneity of asthma it has been a daunting task. Two general approaches have been widely used to study the genetics of asthma: positional cloning and candidate gene approaches. The positional cloning approach in particular has been successful in identifying genes for asthma and asthma-associated phenotypes in recent years.

Multiple genome-wide linkage studies for asthma and allergy have been performed to date. Linkages have been found in specific ethnic groups, using different phenotypes and with various levels of statistical significance. Most of these regions have been replicated in more than one study. In particular, human chromosomes 2q33, 5q23-31, 6p24-21, 11q21-13, 12q24-12, and 13q14-12 have received the greatest attention, because these regions contain a large number of candidate genes [7]. However, most of these identified chromosomal regions are large, spanning 10–30 Mb, and contain several plausible candidate genes. Fine mapping can be performed in these linked regions and positional candidate cloning can be performed using high-throughput sequencing, single nucleotide polymorphism (SNP) genotyping and linkage disequilibrium (LD) mapping. This has enabled researchers to identify susceptibility genes without prior knowledge of the function of those genes e.g. a disintegrin and metalloproteinase-33 (ADAM 33), dipeptidyl dipeptidase-10, plant homeodomain finger protein-11 and G protein-coupled receptor-154 [8].

Association studies can be more powerful than linkage studies in certain circumstances. With the development of high throughput and accurate technologies for DNA sequencing and SNP genotyping the number of asthma candidate genes has increased quickly. Hoffjan *et al*. reviewed 64 candidate genes in 2003 [9] and the number increased to 120 [10] in 2006. There are 53 genes that were reported to be associated with asthma during 2006–2007 (see Additional file 1). Among them filaggrin is notable since seven replication studies were published in independent populations in one year. Another five genes, PTGER3, MYLK, IL17F, ECP and CYSLTR1 were replicated by other groups. The genome-wide association studies currently underway are likely to identify multiple additional genetic variants that are associated with asthma and associated traits.

Following candidate gene identification, it is important to explore the functional consequences of the associated genetic variation. It is likely that many of the genetic variants associated with asthma do not have functional consequences but are simply in LD with the causal variants. For SNPs in coding regions it is possible to predict the consequences for protein structure and function, although such predictions have to be empirically verified. However, most SNPs in the human genome are found in non-coding DNA. Variants located in promoter regions may change gene expression by altering transcription factor binding sites or by other more subtle mechanisms. Intronic variants may have an effect on the alternate splicing pattern in a given cell or tissue. It has been reported that non-coding sequence, which accounts for at least 30% of the human genome, has an important unexpected function since it is a major source of regulatory RNAs in complex organisms [11,12]. In the 2003 review by Hoffjan *et al*. [9] 40 mutations had reports of functional effects and here we list other genes which have functional reports (see Additional file 2).

Recently, another type of genetic variation, structural variations which are mainly found in the form of copy number variants (CNVs), has attracted a large amount of interest from complex disease researchers. Structural variations are widespread in the human genome and may have more functional impact on phenotypic variation than SNPs [13]. The high throughput genotyping of structural variations has become possible. CNVs have already been shown to be associated with susceptibility to HIV infection [14], lupus glomerulonephritis [15], and autoimmune diseases [16,17]. There is no report concerning an association between CNVs and asthma to date but studies are likely underway.

This review focuses on several genes, which provide examples of the investigation of asthma and allergy susceptibility genes.

### Asthma susceptibility genes

#### Filaggrin

Filaggrin (FLG) is a key protein of the epidermis and is therefore important in the formation of the protective skin barrier. *FLG *was initially described as a candidate gene for atopic dermatitis in 2006 [18]. This was a breakthrough discovery because most previous allergy genetics studies were focused on immunological mechanisms and this was the first study to show that genetic variants in the skin defense system are important in allergic pathways. The skin barrier is part of the innate immune system which keeps water within the body and prevents the entrance of pathogens and allergens [19]. With a defective epidermal barrier allergen may more easily gain entrance through the skin and thus initiate local and systemic allergy and predispose to allergic disease.

*FLG *was initially identified as an ichthyosis vulgaris candidate gene in a study of 15 families segregating this single gene disorder [20]. There are three exons in *FLG *and two mutations (R501X and 2282del4) were found in the ichthyosis vulgaris patients. Both mutations are in exon 3 and stop protein translation within the first *FLG *repeat and result in complete loss of FLG peptide production [20]. Therefore, these two functional mutations were considered as the likely causal mutations for ichthyosis vulgaris in these families. Palmer *et al*. [18] observed that many individuals with either of the variations also had atopic dermatitis or asthma and therefore investigated whether the two functional variations also contribute to allergic disease. These authors examined the two mutations in three independent cohorts and found that the results were consistent in that the two nonsense mutations were highly significantly associated with atopic dermatitis (AD) but not with asthma [18]. During the following year these results were replicated by three independent studies, two from Germany [21,22] and one from the UK [23]. In one German study, which included family-based and case-control association analyses, the two mutant alleles showed association with AD as well as asthma in the context of AD but showed no association with asthma or the presence of specific IgE in the absence of AD [22]. Another German study including 476 complete trios (mother, father and affected child) showed association between extrinsic AD but no association with intrinsic AD, the latter defined as dermatitis patients who had normal IgE and lack of sensitization towards environmental allergens [21]. A further study from Germany confirmed the association of *FLG *variants with AD [24]. Another three studies have shown association of extrinsic AD, asthma and rhinitis, asthma severity with the *FLG *variants although in some studies association was only seen with concomitant AD [21,25,26]. All these data suggest that the two *FLG *mutant alleles are important risk factors for AD but only for asthma when it is found in the context of AD. An additional seven nonsense or frame shift *FLG *mutations were identified in the European population and two in the Asian population [27]. There were three reasonably prevalent mutations (in addition to R501X and 2282del4) that showed association with childhood eczema [27].

To date there have been no reports showing lack of association of *FLG *variants and AD. However, the frequency of the two variants is low (less than 5% in Caucasians) and they are absent in Asians and Africans, and therefore only contribute to a small proportion of AD cases [22]. Most AD cases must be caused by other factors and genes that affect skin barrier development are good candidates for further studies.

#### Interleukin-13

Interleukin-13 (*IL13*) is a good candidate as an asthma susceptibility gene because it is a cytokine produced by Th2 cells and because its genetic location on chromosome 5q31 has been linked to asthma and related phenotypes in multiple linkage studies [28-34]. This cytokine is capable of promoting allergen-induced bronchial hyperresponsiveness, epithelial cell damage, goblet cell hyperplasia with mucus hyperproduction, and eosinophilia. There are several studies that have shown that common polymorphisms in this gene, i.e. -1111C > T and +2043G > A (R130Q), are associated with asthma and/or related phenotypes such as increased total serum IgE, atopy, and atopic dermatitis [35-41]. Other investigators have found further evidence for the role of *IL13 *polymorphisms in the pathogenesis of allergic disease [42-46].

Tarazona-Santos *et al. *resequenced the whole *IL13 *gene and confirmed that +2043G > A, which causes replacement of a positively charged arginine (R) with a neutral glutamine (Q) at position 130, was the only nonsynonymous substitution present in all ethnic groups [47]. The 130 position is in the α-helix D segment, which has been proven to be a region where IL13 interacts with IL4 receptor-α/IL13 receptor-α1 heterodimers [48]. In the study of Chen *et al. *the 130Q *IL13 *variant (named Q110R in that paper, because the 20-amino acid signal sequence was not numbered) enhanced IL13-dependent gene induction at the cellular level and induced more significant bronchial hyperresponsiveness in mice compared with the 130R *IL13 *variant [49]. These authors also found that this functional variant had synergistic effects with other functional variants, e.g. 50 V and 551R in the IL4 receptor-α gene [49]. Vladich *et al. *investigated the function of R130Q in peripheral blood mononuclear cells from normal donors [50]. They incubated these cells with the IL13 variants and compared the STAT6 phosphorylation, CD23 expression, and hydrocortisone-dependent IgE synthesis. In these multiple functional assays IL13 130Q showed more activity than wild type IL13 and was less effectively neutralized by soluble IL13 receptor-α. From these results the authors concluded that the +2043G > A polymorphism increased the activity of IL13 and therefore enhanced the pathways leading to allergic inflammation [50]. Both of these papers offered functional evidence demonstrating that the +2043G > A polymorphism plays an important role in the pathogenesis of asthma. None of these results are definitive individually but taken together provide strong evidence that *IL13 *is a susceptibility gene for allergic disease.

Another polymorphism, -1111C > T, is in the *IL13 *promoter region. This promoter SNP shows low levels of LD with +2043G > A in most populations [47]. Therefore, the associations between -1111C > T and asthma-related phenotypes [35,41] are likely independent of the +2043G > A polymorphism. It was observed that the T allele of -1111C > T had increased binding of a T cell transcription factor (NFAT), which regulates *IL13 *and *IL4 *expression and -1111TT homozygosity was associated with both asthma and altered regulation of IL13 production [35]. It is still unclear whether the two polymorphisms act synergistically. However, it is possible that the increased transcription caused by -1111C > T combined with the enhanced activity caused by +2043G > A would amplify the IL13-dependent inflammatory reaction.

#### Interleukin-17F

Unlike IL13, IL17F was discovered recently [51-53]. It is one member of the IL17 gene family and the coding sequence contains 7742 bp, including three exons. IL17F was investigated as an asthma candidate gene because of its function i.e. IL17F is one of the cytokines produced by activated mast cells, CD4+ T cells, and basophils and can upregulate IL6 and IL8 transcripts and protein expression in primary bronchial epithelial cells [51]. IL17F is expressed in human liver, lung, and fetal liver tissue [51] and increased expression was oberserved in the airways of allergic asthma patients [51]. In addition, *IL17F *is located on chromosome 6p, which has been linked to asthma and asthma-related phenotypes in multiple genome scans [54,55].

A Japanese case-control study reported an protective association between homozygosity for the nonsynonymous variant H161R and asthma but no association between heterozygosity for H161R or IL17F haplotypes and asthma [56]. Importantly, there were no homozygotes for 161R in the 432 asthma patients (compared with 9 homozygotes in the 435 controls). Kawaguchi *et al. *[56] also performed experiments to determine whether H161R was a functional variant. Recombinant wild type IL17F (containing histidine at position 161) and mutant IL17F (containing arginine at position 161) were used to stimulate BEAS-2B cells (a human bronchial epithelial cell line) and the results demonstrated that the mutant IL17F could not induce activation of a signaling pathway involving RAF1, MAP2K1/2 and MAPK1/3. The mutant isoform was also unable to stimulate the production of cytokines e.g. CSF2 and chemokines e.g. IL8, CXCL1 and CXCL5. Moreover, the mutant IL17F acted as an antagonist of wild type IL17F to block induction of IL8 expression [56]. However, despite this strong functional data, the association results were not replicated by another case-control study [57]. In this study, the authors genotyped five *IL17F *SNPs including H161R in 1027 white females and found no association between any SNP or haplotype with asthma [57]. The lack of consistency between the two studies may be due to differences in environmental factors or modifier genes that influence the H161R association. Thus, the results of Kawaguchi *et al. *[56] await confirmation in other populations and at the *in vivo *level.

Several additional studies have shed light on the function of IL17F and further suggested that it is a good candidate asthma susceptibility gene. It was reported that *IL17F *was expressed in bronchial epithelial cells and inflammatory cells in an allergic asthma mouse model but not in the lungs of control mice [58]. IL17 induced the expression of IFN-gamma-induced protein 10 (IP10) in bronchial epithelial cells [59]. IP10 has been shown to be a marker of virus-induced asthma [60,61]. In addition, the epigenetic changes in the IL17A-IL17F locus are associated with the differentiation of the novel T helper subset, TH17 cells [62].

#### Cysteinyl leukotriene receptors

Cysteinyl leukotrienes are bronchoconstrictors and proinflammatory mediators of the asthma response that act through two G protein-coupled receptors: cysteinyl leukotriene receptor-1 (CYSLTR1) [63] and CYSLTR2 [64]. Both receptors are the targets of anti-asthmatic drugs. *CYSLTR2 *maps to chromosome 13q14, approximately 300 kb from D13S153, which was reported linked to asthma in two studies [65,66]. The association of *CYSLTR2 *and asthma was replicated in two subsequent studies [67,68] and two functional studies showed potency of leukotriene D4 on the M202V variant was lower compared with the wild-type receptor [68,69].

*CYSLTR1 *is located on chromosome Xq21.1. Five recent studies reported that SNPs in *CYSLTR1 *were associated with allergic phenotypes. Among them, two studies used Caucasian samples and both of them reported significant results of the synonymous SNP, 927T > C [70,71]. A discrepancy between these studies is that the results from the family study [70] showed that the 927T allele had a strong association with atopy severity in female subjects but the results from the case-control study showed that the minor allele, 927C, associated with asthma present with atopic dermatitis but only in males [71]. A study of a Spanish population found that the combination of 927T of *CYSLTR1 *and -444A of the leukotriene C4 synthase gene was less common in male patients with asthma than in controls [72]. *CYSLTR1 *promoter haplotypes were associated with aspirin-sensitive asthma in a Korean population, but again only in male patients [73]. *CYSLTR1 *promoter haplotypes have also been associated with functional effects [73,74]. The 927T > C SNP is in strong LD with the promoter SNPs both in Caucasian and Asian populations in the HapMap database [75]. Consequently, the five studies effectively examined the same haplotype. In the Korean studies, they found that the haplotype containing the minor alleles of the promoter SNPs increased the promoter activity in three cell lines: Jurkat, A549, and U937 cells [73,74]. This result is consistent with another paper in which the U937 cell line was used [76]. Furthermore, at the -475A > C promoter SNP, the A allele was found to bind a specific protein that was not bound by the C allele [74]. However, another study found a contradictory result in THP1 cells [77]. The authors found that the G allele of promoter SNP -336A > G (the minor allele) was associated with a twofold decrease in luciferase expression [77]. The discrepancy may have resulted from the use of different cell lines or the length of the constructs. Therefore, more studies are needed to validate these results.

A further complication is that the precise location of the *CYSLTR1 *promoter region is still unclear. In three papers [73,76,77] 5'RACE was performed using cDNA from human fetus and human leukocytes and no novel exon was found. However, in another paper two novel upstream exons were found before the previously described exon 1 using cDNA from U937 and THP-1 cells and the promoter region was identified in the region between -125 bp to -786 bp upstream of the novel exons [78]. It is possible that there are two alternative promoters in *CYSLTR1 *and they initiate different transcripts. There are no reports of any SNPs in the new promoter region.

#### ORMDL3

In a genome-wide association study performed by Moffatt *et al. *SNPs in the 17q21 region showed a strong association with childhood asthma in both a UK family cohort and German case-control samples. Furthermore, the results were replicated in two independent cohorts [79]. The authors also measured global gene expression in Epstein-Barr virus-transformed lymphoblastoid cells from children in their genotyped family samples and found that the markers which showed strongest association with asthma were also consistently associated with transcript levels of *ORMDL3 *[79]. No other transcript levels were associated with these markers. In this study, the markers which showed association are in different LD blocks so it is possible that multiple functional variants independently contribute to the disease susceptibility. There are also many structural variants in this region that may contribute to the pathogenesis of asthma. This study is remarkable due to the use of genome-wide association analysis coupled with genome-wide gene expression analysis.

*ORMDL3 *is a poorly characterized gene and the underlying mechanism for the association with asthma is unclear. ORMDL3 is a member of a family of endoplasmic reticulum membrane proteins and has a ubiquitous pattern of expression in humans and *Drosophila*. It encodes transmembrane proteins anchored in the endoplasmic reticulum and shows conservation across species [80].

## Conclusion

The pathogenesis of asthma, a complex disease, involves gene-gene interactions as well as gene-environment interactions. Multiple modest risk factors work synergistically to influence asthma disease susceptibility. The number of asthma-susceptibility genes identified by genetic studies is still increasing. However, most studies lack information on the mechanism by which the SNPs lead to asthma. Elucidating the functional consequences of SNPs is essential to confirm association results and to understand how the SNPs combine to influence susceptibility. Eventually, results from this important field are expected to improve preventive strategies and to aid in the development of diagnostic tools and therapies.

## Competing interests

The author(s) declare that they have no competing interests.

## Authors' contributions

JZ drafted the manuscript. PP conceived of the review and edited the manuscript. AS edited the manuscript. All authors read and approved the final manuscript.

## Supplementary Material

Additional file 1 Novel candidate genes in 2006–2007. The table lists all the novel candidate asthma gene studies published during 2006–2007 Click here for file

Additional file 2 Studies to detect functional SNPs. The table lists all the genes with functional studies published during 2005–2007 Click here for file
