# Supplementary material for: Recent advances in asthma genetics
Source: Respir Res. 2008 Jan 15;9(1):4. doi: 10.1186/1465-9921-9-4 (PMC2244620; doi:10.1186/1465-9921-9-4)
Supplement: Additional file 1 — Novel candidate genes in 2006–2007. The table lists all the novel candidate asthma gene studies published during 2006–2007 [file 1465-9921-9-4-S1.doc]

| Gene | Chromosomal location | Gene ID | Population | Sample size | SNP | Phenotype | p value | Influence on function | Ref. |
| --- | --- | --- | --- | --- | --- | --- | --- | --- | --- |
| PTGER3 | 1p31 | 5733 | Korean | Case-control (480/140) | 1388T>C | Asthma | p = 0.002 | unknown | [1] |
|  |  |  | Korean | Case-control (108/140) | -1709T>A | AIA | p =0.043 | unknown | [2] |
| GCLM | 1p22 | 2730 | Russian | Case-control (221/ 214) | -588C/T and -23G/T | Decreased risk of allergic asthma | p = 0.036 | unknown | [3] |
| FLG | 1q21.3 | 2312 | Irish | Case-control (52/189) | R510X | AD | p = 6×10^-9^ | yes | [4] |
|  |  |  |  |  | 2282del4 | AD | p = 8×10^-10^ | yes | [4] |
|  |  |  |  |  | R510X + 2282del4 | AD | p = 3×10^-17^ | yes | [4] |
|  |  |  |  | Case-control (21/189) | R510X + 2282del4 | AD + asthma | p = 6×10^-12^ | yes | [4] |
|  |  |  | Scottish | Case-control (604/1008) | R510X | Asthma | p = 0.024 | yes | [4] |
|  |  |  |  |  | 2282del4 | Asthma | p = 0.00089 | yes | [4] |
|  |  |  |  |  | R510X + 2282del4 | Asthma | p = 6×10^-5^ | yes | [4] |
|  |  |  |  | Case-control (279/1008) | R510X + 2282del4 | AD + asthma | p = 4.8×10^-11^ | yes | [4] |
|  |  |  | Danish | Case-control (142/190) | R510X + 2282del4 | AD | p = 0.006 | yes | [4] |
|  |  |  |  | Case-control (25/190) | R510X + 2282del4 | AD + asthma | p = 0.027 | yes | [4] |
|  |  |  | White | 490 families | R510X + 2282del4 | Eczema | p = 1.9 × 10^−9^ | yes | [5] |
|  |  |  |  |  | R510X + 2282del4 | Eczema + asthma | p = 0.00042 | yes | [5] |
|  |  |  |  |  | R510X + 2282del4 | Eczema + rhinitis; | p = 2.5×10^-5^; | yes | [5] |
|  |  |  |  |  | R510X + 2282del4 | Eczema + specific IgE | p = 1.9×10^-9^ | yes | [5] |
|  |  |  | European | Case-control (170/314) | R510X | Eczema | p = 0.00014 | yes | [5] |
|  |  |  |  | Case-control (172/315) | 2282del4 | Eczema | p = 0.057 | yes | [5] |
|  |  |  |  | Case-control (155/298) | R510X + 2282del4 | Eczema | p = 3.5×10^-5^ | yes | [5] |
|  |  |  |  | Case-control (97/298) | R510X + 2282del4 | Atopic eczema | p = 0.00006 | yes | [5] |
|  |  |  |  | Case-control (52/298) | R510X + 2282del4 | Nonatopic eczema | p = 0.00065 | yes | [5] |
|  |  |  |  | Case-control (30/298) | R510X + 2282del4 | Eczema + asthma | p = 5.4×10^-8^ | yes | [5] |
|  |  |  |  | Case-control (35/298) | R510X + 2282del4 | Eczema + rhinitis | p = 1.5×10^-5^ | yes | [5] |
|  |  |  | German | 476 trios | R510X + 2282del4 | AD | p = 5.1×10^-8^ | yes | [6] |
|  |  |  |  |  | R510X + 2282del4 | Allergic sensitization | p = 2.3×10^-7^ | yes | [6] |
|  |  |  |  |  | R510X + 2282del4 | Total IgE level | p = 9.8×10^-8^ | yes | [6] |
|  |  |  |  |  | R510X + 2282del4 | Asthma | 0.0003 | yes | [6] |
|  |  |  |  |  | R510X + 2282del4 | Extrinsic AD | p = 9.3×10^-8^ | yes | [6] |
|  |  |  |  |  | R510X + 2282del4 | Palmar hyperlinearity | p = 5.9×10^-6^ | yes | [6] |
|  |  |  | White | Case-control (163/1463) | R510X + 2282del4 | AD | p = 1.7×10^-53^ | yes | [7] |
|  |  |  | Japanese | Case-control (143/156) | S2554X + 3321delA | AD | p = 0.0015 | yes | [8] |
|  |  |  | European | Total ( 874) | R501X and 2282del4 | Asthma severity | p = 0.008-0.001 | yes | [9] |
|  |  |  | European | Case-control (188/736) | R501X+ 2282del4+ R2447X+ S3247X+ 3702delG | Childhood eczema | p = 2.12 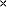10^-51^ | yes | [10] |
|  |  |  | French | Case-control (99/102) | R510X + 2282del4 | AD | p < 0.05 | yes | [11] |
| LELP1 | 1q21 | 149018 | Indian | 119 families | (GT)_n_ | Log_10_ IgE levels | p = 0.0008 | unknown | [12] |
|  |  |  |  | Q: 165 | rs7534334 | Log_10_ IgE levels in the atopic asthmatics | p = 0.0029 | unknown | [12] |
| TGFB2 | 1q41 | 7042 | Japanese | Case-control (297/555) | 94862T >A; -109-->ACAA ins | AD | p = 0.0037-0.00041 | Yes ( -109-->ACAA ins) | [13] |
| PPARG | 3p25 | 5468 | Caucasian | Q: 569 | Pro12Ala; C1431T | Asthma exacerbations | p = 0.006-0.036 | unknown | [14] |
|  |  |  |  |  | Haplotype | Increased risk for asthma exacerbations | p = 0.002 | unknown | [14] |
| CCR2 | 3p21 | 1231 | Korean | Case-control (65/1366) | Ile64Val | Asthma | p = 0.04 | unknown | [15] |
| CX3CR1 | 3p21.3 | 1524 | French Canadian | 223 families | rs938203, rs2669849, rs1050592, T280M, V249I | Asthma | p < 0.004 | unknown | [16] |
|  |  |  |  | 223 families | Haplotype | Asthma | p = 0.005 | unknown | [16] |
|  |  |  | Multiple populations | Case-control (178/268) | rs2669849 | Asthma | p = 0.0073 | unknown | [16] |
|  |  |  |  | Case-control (174/267) | V249I | Asthma | p = 0.031 | unknown | [16] |
| CSTA | 3q21 | 1475 | Caucasian | Case-control (100/264) | C+344T | AD | p = 0.006 | yes | [17] |
| MYLK | 3q21 | 4638 | African American | Case-control (102/93) | Pro147Ser | Severe asthma | p = 0.037 | unknown | [18] |
|  |  |  | African American | 125 African Caribbean families | Haplotype | Decreased risk of asthma | p = .004 | unknown | [19] |
|  |  |  |  | 89 African American families | Haplotype | Decreased risk of asthma | p = .005 | unknown | [19] |
| CD86 | 3q21 | 942 | Danish | 235 families | Ile179Val | Asthma | p = 4x10^–3^ | yes | [20] |
| COL29A1 | 3q22 | 256076 | German | 199 families + 292 families | Haplotype | AD | p = 0.000059 | unknown | [21] |
| PDGFRA | 4q11-q13 | 5156 | Chinese | Case-control (277/93) | rs1800810 | Nonallergic asthma | p = 0.038 | unknown | [22] |
|  |  |  |  |  | Haplotype | Nonallergic asthma | p < 0.02 | unknown | [22] |
|  |  |  |  |  | Haplotype | Persistent asthma | p = 0.008 | unknown | [22] |
|  |  |  |  |  | Haplotype | Low PDGF-AA serum level | p < 0.05 | unknown | [22] |
| VEGFR2 | 4q11-q12 | 3791 | Korean | Case-control (761/1296) | V297I | Atopy | p = 0.048 | unknown | [23] |
|  |  |  |  | Case-control (761/1296) | Haplotype | Atopy | p = 0.002 | unknown | [23] |
| CXCL9 | 4q21 | 4283 | Japanese | 48 families | rs2869460 | Allergic Rhinitis | p = 0.04 | unknown | [24] |
|  |  |  | Japanese | 48 families | rs2276886 | Allergic Rhinitis | p = 0.014 | unknown | [24] |
| CXCL10 | 4q21 | 3627 | Japanese | 48 families | rs2869462 | Allergic Rhinitis | p = 0.03 | unknown | [24] |
| CXCL11 | 4q21 | 6373 | Japanese | 48 families | rs12649185 | Allergic Rhinitis | p = 0.03 | unknown | [24] |
| SPP1 | 4q22 | 6696 | Japanese | Q : 343 | 5891C/T | Increased total serum IgE | p = 0.009 | unknown | [25] |
|  |  |  |  | Q : 343 | 7052T/C | Increased total serum IgE | p = 0.001 | unknown | [25] |
| GSNOR | 4q23 | 128 | Mexican-American | 532 families | rs28730619 | Asthma | p = 0.0077 | unknown | [26] |
|  |  |  |  | 532 families | rs1154404 | Decreased risk of asthma | p = 0.028 | unknown | [26] |
| IL2 | 4q27 | 3558 | Danish | 235 families | rs2069762 | Asthma; PRT; Rhinitis; Atopic Dermatitis | p < 0.05 | yes | [27] |
|  |  |  |  | 235 families | rs2069763 | PRT; PST | p < 0.05 | unknown | [27] |
|  |  |  |  | 235 families | Haplotypes | Asthma; PRT; PST; Rhinitis; Atopic Dermatitis | p < 0.05 | unknown | [27] |
| PTGER4 | 5p13 | 5734 | Korean | Case-control ( 108/140) | -1254A>G | AIA | p =0.018 | unknown | [2] |
| IL9 | 5q31.1 | 3578 | Taiwanese | 30 trios | GT repeat | Asthma with HD | p = 0.038 | unknown | [28] |
|  |  |  |  | 69 trios | GT repeat | Asthma with Der p (or Der f) | p =0.03 | unknown | [28] |
|  |  |  | Taiwanese | 123 families | GT repeat | Atopic asthma | p = 0.03 | unknown | [28] |
| DCNP1 | 5q31 | 140947 | Korean | Case-control (1044/ 287) | c.-1289C>T | Specific IgE | p = 0.0009-0.04 | yes | [29] |
| TIM4 | 5q33.3 | 91937 | Caucasian | 93 families | Haplotype | AD | p = 0.016 | unknown | [30] |
| ITK | 5q31-q32 | 3702 | Caucasian and Hispanic | Total (564) | rs451494 | Atopy | p < 0.05 | unknown | [31] |
|  |  |  | Caucasian and Hispanic | Total (564) | rs365171 | Atopy | p < 0.05 | unknown | [31] |
| IL17F | 6p12 | 112744 | Japanese | Case-control (432/435) | rs763780 | Asthma | p = 0.0079 | Yes | [32] |
|  |  |  | Japanese | Case-control (total 690/435) | rs763780 | Asthma + COPD | p < 0.005 | Yes | [33] |
| MICB | 6p21.3 | 4277 | Australian | Case-control (136/339) | AluyMICB DD | Asthma in men | p = 0.006 | unknown | [34] |
|  |  |  |  | Case-control (222/412) | AluyMICB II | FEV1 in women | p = 0.001 | unknown | [34] |
| ESR1 | 6q25.1 | 2099 | Dutch | Family base study (1249 individuals) | IVS1−397C/T | BHR / Female BHR | p = 0.02 / 0.01 | unknown | [35] |
|  |  |  |  | 200 Asthmatics | IVS1−1505A/G; IVS1−1415T/C; IVS1−397C/T; IVS1−351G/A; exon1+30T/C | FEV_1_ decline | p = 0.01-0.04 | unknown | [35] |
| FYN | 6q21 | 2534 | Polish | Case-control (120/187) | -93A/G; Ex12+894T/G | Asthma | p = 0.019-0.024 | unknown | [36] |
| AOAH | 7p14-p12 | 313 | African | 125 families | Multiple SNPs | Asthma; Log (IgE);log[IL-13]/log[IFN-γ]; sCD14 | p < 0.05 | unknown | [37] |
|  |  |  |  |  | Haplotype | Log (IgE) | p = 0.006 | unknown | [37] |
| SFTPC | 8p21 | 6440 | German | Case-control (131/270) | Haplotypes | Severe respiratory syncytial virus infection | p = 0.013 | unknown | [38] |
| RIP2 | 8q21 | 8767 | Japanese | Case only study(300) | -980T/G | Childhood severe asthma | p = 0.0032 | unknown | [39] |
| NK2R | 10q11-q21 | 6865 | Korean | Q : 70 | 7853G>A | High serum vascular endothelial growth factor | p = 0.040 | unknown | [40] |
| PLAU | 10q24 | 5328 | French-Canadian | 231 families | rs2227564, rs2227566, haplotype | Asthma; AHR; Atopy | p = 0.006 -0.045 | unknown | [41] |
|  |  |  | Multiple population | 237 trios | rs4065 | Atopy | p = 0.0001 | unknown | [41] |
| CAT | 11p13 | 847 | Chinese | Case-control (251/316) | C-262T | Asthma | p = 0.033 | unknown | [42] |
| BDNF | 11p13 | 627 | Polish | Case-control (56/109) | -270C/T | Asthma | p = 0.005 | unknown | [43] |
| IRAKM | 12q14 | 11213 | Sardinian founder population | 294 families | Haplotype | Early-onset persistent asthma | p =.0011 | unknown | [44] |
|  |  |  |  | Case-control ( 139/ 460) | Haplotype | Early-onset persistent asthma | p =.0081 | unknown | [44] |
|  |  |  | European | Case-control ( 67/ 278) | rs1624395, rs1370128 | Asthma | p = 0.002-0.004 | unknown | [44] |
| LTA4H | 12q22 | 4048 | White | Total 61 asthmatic patients | rs2660845 | Asthma exacerbation rates | p = 0.021 | unknown | [45] |
| SFRS8 | 12q24.33 | 6433 | Danish | 212 families | rs1051219 | Asthma | p = 0.043 | unknown | [46] |
|  |  |  | Danish | 212 families | rs1051233 | Asthma | p = 0.018 | unknown | [46] |
|  |  |  | Danish | 212 families | rs755437 | Asthma | p = 0.02 | unknown | [46] |
| ECP | 14q24-q31 | 6037 | Japanese | Q: 192 asthmatics | -393C/T | Serum ECP levels | p = 0.004 | yes | [47] |
|  |  |  | European | 177 families | Haplotype | Asthma | p = 0.004 | unknown | [48] |
|  |  |  |  |  | Haplotype | High s-ECP, allergic asthma, high s-IgE, and BHR | p = 0.004-0.04 | unknown | [48] |
| IGHG | 14q32.3 | IGHG3: 3502  IGHG1: 3500  IGHG2 : 3501  IGHG4 : 147130 | Swedish | Q: 55 Childhood asthmatics | IGHG*bf/*bf | Higher asthma scores, lower airway function, greater bronchodilator responses, greater AHR and highest specific IgE levels | p = 0.035-0.001 | unknown | [49] |
| IL16 | 15q26.3 | 3603 | White | 341 families | −295 T/C | Asthma | p = 0.015-0.002 | yes | [50] |
| SOCS1 | 16p13.13 | 8651 | Japanese | Case-control (462/639) | -1478CA>del | Adult asthma | p = 0.0063 | yes | [51] |
|  |  |  |  |  | Haplotype | Adult asthma | p = 0.0097 | unknown | [51] |
| MRP1 | 16p13.1 | 4363 | White | Total 61 asthmatic patients | rs119774 | Changes in FEV1 | p = 0.004 | unknown | [45] |
| IL27 | 16p11 | 246778 | Korean | Case-control (288/444) | g.-964A > G | Asthma; Atopy | p = 0.003-0.013 | unknown | [52] |
| ORMDL3 | 17q12-q21 | 94103 | English + German | Family study +case-control (total 2642) | rs9303277; rs11557467; rs8067378; rs2290400; rs7216389; rs4795405; rs8079416; rs4795408; rs3894194; rs3859192 | Asthma | The strongest p < 10^-12^ | unknown | [53] |
|  |  |  | German | case-control (200/2120) | Same SNPs as above | Asthma | p < 0.001 | unknown | [53] |
|  |  |  | Caucasian | case-control (398/2903) | rs3894194 | Asthma | p = 0.012 | unknown | [53] |
| FCER2 | 19p13.3 | 2208 | White and African American children | Q: 311 asthmatics | T2206C | Severe exacerbations and decreased *FCER2* expression in asthmatics | p = 0.02 | unknown | [54] |
| PTGIR | 19q13 | 5739 | Korean | Case-control ( 108/140) | 1915T>C | AIA | p = 0.015 | unknown | [2] |
|  |  |  |  | Case-control ( 108/93/140) | Haplotype | AIA; ATA | p = 0.031-0.047 | unknown | [2] |
| MMP9 | 20q11.2-q13.1 | 4318 | Japanese | Case-control (290/638) | 2127G/T | Childhood atopic asthma | p = 0.0032 | Yes ( Function of SNPs in LD -1590C/T; -1831T/A) | [55] |
|  |  |  |  | Case-control (290/638) | 5546G/A | Childhood atopic asthma | p = 0.0016 | unknown | [55] |
|  |  |  |  | Case-control (290/638) | Haplotype | Childhood atopic asthma | p = 0.0053 | unknown | [55] |
| CD40 | 20q12-q13.2 | 958 | Korean | Q : 478 asthmatics | -580G >A | Total serum IgE levels in asthmatics | p = 0.007 | No function | [56] |
|  |  |  |  | Q : 478 asthmatics | -1C>T | Total serum IgE levels in asthmatics | p = 0.005 | yes | [56] |
| CYP24A1 | 20q13 | 1591 | Germany and Sweden | 224 families | Haplotype | Asthma; Total IgE; calcidiol; calcitriol | p = 0.0046-0.00063 | unknown | [57] |
| RUNX1 | 21q22.3 | 861 | Korean | Case-control (1055/384) | -208G/C | Total IgE | p = 0.03 | unknown | [58] |
|  |  |  |  | Case-control (1055/384) | -1282G/A | Total IgE | p = 0.03 | unknown | [58] |
| CYSLTR1 | Xq13.2-21.1 | 10800 | Korean | Case-control (39/46) | Haplotype | Male AIA | p = 0.03 | yes | [59] |
|  |  |  | Korean | Case-control (39/50) | Haplotype | Male AIA | p = 0.02 | yes | [59] |
|  |  |  | Caucasian | 341 families | 927T/C | Atopy severity | p = 0.0148 | unknown | [60] |
|  |  |  | Caucasian | Case-control (53/24) | 927T/C | AA-AD | p < 0.005 | unknown | [61] |
|  |  |  | Korean | Case-control ( 159/ 116 ) | Haplotype | AIA | p = 0.015 | yes | [62] |
|  |  |  | Tristan da Cunha | Case-control ( Total 112 ) | G300S | Asthma and atopy | p = 0.04-0.001 | yes | [63] |

PTGER3: prostaglandin E receptor 3; GCLM: glutamate-cysteine ligase, modifier subunit; FLG: filaggrin; LELP1: late cornified envelope-like proline-rich 1; TGFB2: transforming growth factor, beta 2; PPARG: peroxisome proliferator-activated receptor gamma; CCR2: chemokine (C-C motif) receptor 2; CX3CR1: chemokine (C-X3-C motif) receptor 1; CSTA: cystatin A; MYLK: myosin, light chain kinase; CD86: CD86 molecule; COL29A1: collagen XXIX alpha 1; PDGFRA: platelet-derived growth factor receptor, alpha polypeptide; VEGFR: kinase insert domain receptor; CXCL9: chemokine (C-X-C motif) ligand 9; CXCL10: chemokine (C-X-C motif) ligand 10; CXCL11: chemokine (C-X-C motif) ligand 11; SPP1: secreted phosphoprotein 1; GSNOR: alcohol dehydrogenase 5; IL2: interleukin 2; PTGER4: prostaglandin E receptor 4; IL9: interleukin 9; DCNP1: chromosome 5 open reading frame 20; TIM4: T-cell immunoglobulin and mucin domain containing; ITK: IL2-inducible T-cell kinase; IL17F: interleukin 17F; MICB: MHC class I polypeptide-related sequence B; ESR: estrogen receptor 1; FYN: FYN oncogene related to SRC; AOAH: acyloxyacyl hydrolase; SFTPC: surfactant, pulmonary-associated protein C; RIP2: receptor-interacting serine-threonine kinase 2; NK2R: tachykinin receptor 2; PLAU: plasminogen activator, urokinase; CAT: catalase; BDNF: brain-derived neurotrophic factor; IRAKM: interleukin-1 receptor-associated kinase 3; LTA4H: leukotriene A4 hydrolase; SFRS8: splicing factor, arginine/serine-rich 8; ECP: eosinophil cationic protein; IGHG: immunoglobulin heavy constant gamma; IL16: interleukin 16; SOCS1: suppressor of cytokine signaling 1; MRP1: Multidrug resistance-associated protein-1; IL27: interleukin 27; ORMDL3: ORM1-like 3; FCER2: Fc fragment of IgE, low affinity II; PTGIR: prostaglandin I2 (prostacyclin) receptor; MMP9: matrix metallopeptidase 9; CD40: CD40 molecule; CYP24A1: cytochrome P450, family 24, subfamily A, polypeptide 1; RUNX1: runt-related transcription factor 1; CYSLTR1: cysteinyl leukotriene receptor 1

AD: Atopic dermatitis; AHR: Airway Hyperresponsiveness; PRT: Positive RAST test; PST: Positive skin prick test; HD: House dust; COPD: Chronic obstructive pulmonary disease; FEV1: Forced expiratory volume in one second; AIA: Aspirin-intolerant asthma; ATA: aspirin-tolerant asthma; BHR: Bronchial hyperresponsiveness; Q: quantitative phenotype; AA-AD: Asthma presented atopic dermatitis

* SNP name used in the table is from the original paper

1. Park HW, Shin ES, Lee JE, Kim SH, Kim SS, Chang YS, Kim YK, Min KU, Kim YY, Cho SH: **Association between genetic variations in prostaglandin E2 receptor subtype EP3 gene (Ptger3) and asthma in the Korean population**. *Clin Exp Allergy* 2007, **37:**1609-1615.

2. Kim SH, Kim YK, Park HW, Jee YK, Bahn JW, Chang YS, Ye YM, Shin ES, Lee JE, Park HS, Min KU: **Association between polymorphisms in prostanoid receptor genes and aspirin-intolerant asthma**. *Pharmacogenet Genomics* 2007, **17:**295-304.

3. Polonikov AV, Ivanov VP, Solodilova MA, Khoroshaya IV, Kozhuhov MA, Panfilov VI: **The relationship between polymorphisms in the glutamate cysteine ligase gene and asthma susceptibility**. *Respir Med* 2007, **101:**2422-2424.

4. Palmer CN, Irvine AD, Terron-Kwiatkowski A, Zhao Y, Liao H, Lee SP, Goudie DR, Sandilands A, Campbell LE, Smith FJ, O'Regan GM, Watson RM, Cecil JE, Bale SJ, Compton JG, DiGiovanna JJ, Fleckman P, Lewis-Jones S, Arseculeratne G, Sergeant A, Munro CS, El Houate B, McElreavey K, Halkjaer LB, Bisgaard H, Mukhopadhyay S, McLean WH: **Common loss-of-function variants of the epidermal barrier protein filaggrin are a major predisposing factor for atopic dermatitis**. *Nat Genet* 2006, **38:**441-446.

5. Marenholz I, Nickel R, Ruschendorf F, Schulz F, Esparza-Gordillo J, Kerscher T, Gruber C, Lau S, Worm M, Keil T, Kurek M, Zaluga E, Wahn U, Lee YA: **Filaggrin loss-of-function mutations predispose to phenotypes involved in the atopic march**. *J Allergy Clin Immunol* 2006, **118:**866-871.

6. Weidinger S, Illig T, Baurecht H, Irvine AD, Rodriguez E, Diaz-Lacava A, Klopp N, Wagenpfeil S, Zhao Y, Liao H, Lee SP, Palmer CN, Jenneck C, Maintz L, Hagemann T, Behrendt H, Ring J, Nothen MM, McLean WH, Novak N: **Loss-of-function variations within the filaggrin gene predispose for atopic dermatitis with allergic sensitizations**. *J Allergy Clin Immunol* 2006, **118:**214-219.

7. Barker JN, Palmer CN, Zhao Y, Liao H, Hull PR, Lee SP, Allen MH, Meggitt SJ, Reynolds NJ, Trembath RC, McLean WH: **Null Mutations in the Filaggrin Gene (FLG) Determine Major Susceptibility to Early-Onset Atopic Dermatitis that Persists into Adulthood**. *J Invest Dermatol* 2006,

8. Nomura T, Sandilands A, Akiyama M, Liao H, Evans AT, Sakai K, Ota M, Sugiura H, Yamamoto K, Sato H, Palmer CN, Smith FJ, McLean WH, Shimizu H: **Unique mutations in the filaggrin gene in Japanese patients with ichthyosis vulgaris and atopic dermatitis**. *J Allergy Clin Immunol* 2007, **119:**434-440.

9. Palmer CN, Ismail T, Lee SP, Terron-Kwiatkowski A, Zhao Y, Liao H, Smith FJ, McLean WH, Mukhopadhyay S: **Filaggrin null mutations are associated with increased asthma severity in children and young adults**. *J Allergy Clin Immunol* 2007, **120:**64-68.

10. Sandilands A, Terron-Kwiatkowski A, Hull PR, O'Regan GM, Clayton TH, Watson RM, Carrick T, Evans AT, Liao H, Zhao Y, Campbell LE, Schmuth M, Gruber R, Janecke AR, Elias PM, van Steensel MA, Nagtzaam I, van Geel M, Steijlen PM, Munro CS, Bradley DG, Palmer CN, Smith FJ, McLean WH, Irvine AD: **Comprehensive analysis of the gene encoding filaggrin uncovers prevalent and rare mutations in ichthyosis vulgaris and atopic eczema**. *Nat Genet* 2007, **39:**650-654.

11. Hubiche T, Ged C, Benard A, Leaute-Labreze C, McElreavey K, de Verneuil H, Taieb A, Boralevi F: **Analysis of SPINK 5, KLK 7 and FLG Genotypes in a French Atopic Dermatitis Cohort**. *Acta Derm Venereol* 2007, **87:**499-505.

12. Sharma M, Mehla K, Batra J, Ghosh B: **Association of a chromosome 1q21 locus in close proximity to a late cornified envelope-like proline-rich 1 (LELP1) gene with total serum IgE levels**. *J Hum Genet* 2007, **52:**378-383.

13. Hatsushika K, Hirota T, Harada M, Sakashita M, Kanzaki M, Takano S, Doi S, Fujita K, Enomoto T, Ebisawa M, Yoshihara S, Sagara H, Fukuda T, Masuyama K, Katoh R, Matsumoto K, Saito H, Ogawa H, Tamari M, Nakao A: **Transforming growth factor-beta(2) polymorphisms are associated with childhood atopic asthma**. *Clin Exp Allergy* 2007, **37:**1165-1174.

14. Palmer CN, Doney AS, Ismail T, Lee SP, Murrie I, Macgregor DF, Mukhopadhyay S: **PPARG locus haplotype variation and exacerbations in asthma**. *Clin Pharmacol Ther* 2007, **81:**713-718.

15. Kim YK, Oh HB, Lee EY, Gho YS, Lee JE, Kim YY: **Association between a genetic variation of CC chemokine receptor-2 and atopic asthma**. *Allergy* 2007, **62:**208-209.

16. Tremblay K, Lemire M, Provost V, Pastinen T, Renaud Y, Sandford AJ, Laviolette M, Hudson TJ, Laprise C: **Association study between the CX3CR1 gene and asthma**. *Genes Immun* 2006, 7:632-639.

17. Vasilopoulos Y, Cork MJ, Teare D, Marinou I, Ward SJ, Duff GW, Tazi-Ahnini R: **A nonsynonymous substitution of cystatin A, a cysteine protease inhibitor of house dust mite protease, leads to decreased mRNA stability and shows a significant association with atopic dermatitis**. *Allergy* 2007, **62:**514-519.

18. Flores C, Ma SF, Maresso K, Ober C, Garcia JG: **A variant of the myosin light chain kinase gene is associated with severe asthma in African Americans**. *Genet Epidemiol* 2007, **31:**296-305.

19. Gao L, Grant AV, Rafaels N, Stockton-Porter M, Watkins T, Gao P, Chi P, Munoz M, Watson H, Dunston G, Togias A, Hansel N, Sevransky J, Maloney JP, Moss M, Shanholtz C, Brower R, Garcia JG, Grigoryev DN, Cheadle C, Beaty TH, Mathias RA, Barnes KC: **Polymorphisms in the myosin light chain kinase gene that confer risk of severe sepsis are associated with a lower risk of asthma**. *J Allergy Clin Immunol* 2007, **119:**1111-1118.

20. Corydon TJ, Haagerup A, Jensen TG, Binderup HG, Petersen MS, Kaltoft K, Vestbo J, Kruse TA, Borglum AD: **A functional CD86 polymorphism associated with asthma and related allergic disorders**. *J Med Genet* 2007, **44:**509-515.

21. Soderhall C, Marenholz I, Kerscher T, Ruschendorf F, Esparza-Gordillo J, Worm M, Gruber C, Mayr G, Albrecht M, Rohde K, Schulz H, Wahn U, Hubner N, Lee YA: **Variants in a novel epidermal collagen gene (COL29A1) are associated with atopic dermatitis**. *PLoS Biol* 2007, **5:**e242.

22. Wu LS, Tan CY, Wang LM, Lin CG, Wang JY: **Variant in promoter region of platelet-derived growth factor receptor-alpha (PDGFRalpha) gene is associated with the severity and allergic status of childhood asthma**. *Int Arch Allergy Immunol* 2006, **141:**37-46.

23. Park HW, Lee JE, Shin ES, Lee JY, Bahn JW, Oh HB, Oh SY, Cho SH, Moon HB, Min KU, Elias JA, Kim YY, Kim YK: **Association between genetic variations of vascular endothelial growth factor receptor 2 and atopy in the Korean population**. *J Allergy Clin Immunol* 2006, **117:**774-779.

24. Zhang J, Noguchi E, Migita O, Yokouchi Y, Nakayama J, Shibasaki M, Arinami T: **Association of a haplotype block spanning SDAD1 gene and CXC chemokine genes with allergic rhinitis**. *J Allergy Clin Immunol* 2005, **115:**548-554.

25. Tanino Y, Hizawa N, Konno S, Fukui Y, Takahashi D, Maeda Y, Huang SK, Nishimura M: **Sequence variants of the secreted phosphoprotein 1 gene are associated with total serum immunoglobulin E levels in a Japanese population**. *Clin Exp Allergy* 2006, **36:**219-225.

26. Wu H, Romieu I, Sienra-Monge JJ, Estela Del Rio-Navarro B, Anderson DM, Jenchura CA, Li H, Ramirez-Aguilar M, Del Carmen Lara-Sanchez I, London SJ: **Genetic variation in S-nitrosoglutathione reductase (GSNOR) and childhood asthma**. *J Allergy Clin Immunol* 2007, **120:**322-328.

27. Christensen U, Haagerup A, Binderup HG, Vestbo J, Kruse TA, Borglum AD: **Family based association analysis of the IL2 and IL15 genes in allergic disorders**. *Eur J Hum Genet* 2006, **14:**227-235.

28. Wang TN, Chen WY, Huang YF, Shih NH, Feng WW, Tseng HI, Lee CH, Ko YC: **The synergistic effects of the IL-9 gene and environmental exposures on asthmatic Taiwanese families as determined by the transmission/disequilibrium test**. *Int J Immunogenet* 2006, **33:**105-110.

29. Kim Y, Park CS, Shin HD, Choi JW, Cheong HS, Park BL, Choi YH, Jang AS, Park SW, Lee YM, Lee EJ, Park SG, Lee JY, Lee JK, Han BG, Oh B, Kimm K: **A promoter nucleotide variant of the dendritic cell-specific DCNP1 associates with serum IgE levels specific for dust mite allergens among the Korean asthmatics**. *Genes Immun* 2007, **8:**369-378.

30. Page NS, Jones G, Stewart GJ: **Genetic association studies between the T cell immunoglobulin mucin (TIM) gene locus and childhood atopic dermatitis**. *Int Arch Allergy Immunol* 2006, **141:**331-336.

31. Graves PE, Siroux V, Guerra S, Klimecki WT, Martinez FD: **Association of atopy and eczema with polymorphisms in T-cell immunoglobulin domain and mucin domain-IL-2-inducible T-cell kinase gene cluster in chromosome 5 q 33**. *J Allergy Clin Immunol* 2005, **116:**650-656.

32. Kawaguchi M, Takahashi D, Hizawa N, Suzuki S, Matsukura S, Kokubu F, Maeda Y, Fukui Y, Konno S, Huang SK, Nishimura M, Adachi M: **IL-17F sequence variant (His161Arg) is associated with protection against asthma and antagonizes wild-type IL-17F activity**. *J Allergy Clin Immunol* 2006, **117:**795-801.

33. Hizawa N, Kawaguchi M, Huang SK, Nishimura M: **Role of interleukin-17F in chronic inflammatory and allergic lung disease**. *Clin Exp Allergy* 2006, **36:**1109-1114.

34. Hui J, Palmer LJ, James AL, Musk AW, Beilby JP: **AluyMICB dimorphism within the class I region of the major histocompatibility complex is associated with asthma and airflow obstruction in the Busselton population**. *Clin Exp Allergy* 2006, **36:**728-734.

35. Dijkstra A, Howard TD, Vonk JM, Ampleford EJ, Lange LA, Bleecker ER, Meyers DA, Postma DS: **Estrogen receptor 1 polymorphisms are associated with airway hyperresponsiveness and lung function decline, particularly in female subjects with asthma**. *J Allergy Clin Immunol* 2006, **117:**604-611.

36. Szczepankiewicz A, Breborowicz A, Skibinska M, Wilkosc M, Tomaszewska M, Hauser J: **Association Analysis of Tyrosine Kinase FYN Gene Polymorphisms in Asthmatic Children**. *Int Arch Allergy Immunol* 2007, **145:**43-47.

37. Barnes KC, Grant A, Gao P, Baltadjieva D, Berg T, Chi P, Zhang S, Zambelli-Weiner A, Ehrlich E, Zardkoohi O, Brummet ME, Stockton M, Watkins T, Gao L, Gittens M, Wills-Karp M, Cheadle C, Beck LA, Beaty TH, Becker KG, Garcia JG, Mathias RA: **Polymorphisms in the novel gene acyloxyacyl hydroxylase (AOAH) are associated with asthma and associated phenotypes**. *J Allergy Clin Immunol* 2006, **118:**70-77.

38. Puthothu B, Krueger M, Heinze J, Forster J, Heinzmann A: **Haplotypes of surfactant protein C are associated with common paediatric lung diseases**. *Pediatr Allergy Immunol* 2006, **17:**572-577.

39. Nakashima K, Hirota T, Suzuki Y, Akahoshi M, Shimizu M, Jodo A, Doi S, Fujita K, Ebisawa M, Yoshihara S, Enomoto T, Shirakawa T, Kishi F, Nakamura Y, Tamari M: **Association of the RIP2 gene with childhood atopic asthma**. *Allergol Int* 2006, **55:**77-83.

40. Ye YM, Kang YM, Kim SH, Kim CW, Kim HR, Hong CS, Park CS, Kim HM, Nahm DH, Park HS: **Relationship between neurokinin 2 receptor gene polymorphisms and serum vascular endothelial growth factor levels in patients with toluene diisocyanate-induced asthma**. *Clin Exp Allergy* 2006, **36:**1153-1160.

41. Begin P, Tremblay K, Daley D, Lemire M, Claveau S, Salesse C, Kacel S, Montpetit A, Becker A, Chan-Yeung M, Kozyrskyj AL, Hudson TJ, Laprise C: **Association of urokinase-type plasminogen activator with asthma and atopy**. *Am J Respir Crit Care Med* 2007, **175:**1109-1116.

42. Mak JC, Leung HC, Ho SP, Ko FW, Cheung AH, Ip MS, Chan-Yeung MM: **Polymorphisms in manganese superoxide dismutase and catalase genes: functional study in Hong Kong Chinese asthma patients**. *Clin Exp Allergy* 2006, **36:**440-447.

43. Szczepankiewicz A, Breborowicz A, Skibinska M, Wilkosc M, Tomaszewska M, Hauser J: **Association analysis of brain-derived neurotrophic factor gene polymorphisms in asthmatic children**. *Pediatr Allergy Immunol* 2007, **18:**293-297.

44. Balaci L, Spada MC, Olla N, Sole G, Loddo L, Anedda F, Naitza S, Zuncheddu MA, Maschio A, Altea D, Uda M, Pilia S, Sanna S, Masala M, Crisponi L, Fattori M, Devoto M, Doratiotto S, Rassu S, Mereu S, Giua E, Cadeddu NG, Atzeni R, Pelosi U, Corrias A, Perra R, Torrazza PL, Pirina P, Ginesu F, Marcias S, Schintu MG, Del Giacco GS, Manconi PE, Malerba G, Bisognin A, Trabetti E, Boner A, Pescollderungg L, Pignatti PF, Schlessinger D, Cao A, Pilia G: **IRAK-M is involved in the pathogenesis of early-onset persistent asthma**. *Am J Hum Genet* 2007, **80:**1103-1114.

45. Lima JJ, Zhang S, Grant A, Shao L, Tantisira KG, Allayee H, Wang J, Sylvester J, Holbrook J, Wise R, Weiss ST, Barnes K: **Influence of leukotriene pathway polymorphisms on response to montelukast in asthma**. *Am J Respir Crit Care Med* 2006, **173:**379-385.

46. Brasch-Andersen C, Tan Q, Borglum AD, Haagerup A, Larsen TR, Vestbo J, Kruse TA: **Significant linkage to chromosome 12q24.32-q24.33 and identification of SFRS8 as a possible asthma susceptibility gene**. *Thorax* 2006, **61:**874-879.

47. Noguchi E, Iwama A, Takeda K, Takeda T, Kamioka M, Ichikawa K, Akiba T, Arinami T, Shibasaki M: **The promoter polymorphism in the eosinophil cationic protein gene and its influence on the serum eosinophil cationic protein level**. *Am J Respir Crit Care Med* 2003, **167:**180-184.

48. Munthe-Kaas MC, Gerritsen J, Carlsen KH, Undlien D, Egeland T, Skinningsrud B, Torres T, Carlsen KL: **Eosinophil cationic protein (ECP) polymorphisms and association with asthma, s-ECP levels and related phenotypes**. *Allergy* 2007, **62:**429-436.

49. Gustafsson PM, Oxelius VA, Nilsson S, Kjellman B: **Association between Gm allotypes and asthma severity from childhood to young middle age**. *Respir Med* 2007,

50. Burkart KM, Barton SJ, Holloway JW, Yang IA, Cakebread JA, Cruikshank W, Little F, Jin X, Farrer LA, Clough JB, Keith TP, Holgate S, Center DM, O'Connor GT: **Association of asthma with a functional promoter polymorphism in the IL16 gene**. *J Allergy Clin Immunol* 2006, **117:**86-91.

51. Harada M, Nakashima K, Hirota T, Shimizu M, Doi S, Fujita K, Shirakawa T, Enomoto T, Yoshikawa M, Moriyama H, Matsumoto K, Saito H, Suzuki Y, Nakamura Y, Tamari M: **Functional polymorphism in the suppressor of cytokine signaling 1 gene associated with adult asthma**. *Am J Respir Cell Mol Biol* 2007, **36:**491-496.

52. Chae SC, Li CS, Kim KM, Yang JY, Zhang Q, Lee YC, Yang YS, Chung HT: **Identification of polymorphisms in human interleukin-27 and their association with asthma in a Korean population**. *J Hum Genet* 2007, **52:**355-361.

53. Moffatt MF, Kabesch M, Liang L, Dixon AL, Strachan D, Heath S, Depner M, von Berg A, Bufe A, Rietschel E, Heinzmann A, Simma B, Frischer T, Willis-Owen SA, Wong KC, Illig T, Vogelberg C, Weiland SK, von Mutius E, Abecasis GR, Farrall M, Gut IG, Lathrop GM, Cookson WO: **Genetic variants regulating ORMDL3 expression contribute to the risk of childhood asthma**. *Nature* 2007, **448:**470-473.

54. Tantisira KG, Silverman ES, Mariani TJ, Xu J, Richter BG, Klanderman BJ, Litonjua AA, Lazarus R, Rosenwasser LJ, Fuhlbrigge AL, Weiss ST: **FCER2: A pharmacogenetic basis for severe exacerbations in children with asthma**. *J Allergy Clin Immunol* 2007,

55. Nakashima K, Hirota T, Obara K, Shimizu M, Doi S, Fujita K, Shirakawa T, Enomoto T, Yoshihara S, Ebisawa M, Matsumoto K, Saito H, Suzuki Y, Nakamura Y, Tamari M: **A functional polymorphism in MMP-9 is associated with childhood atopic asthma**. *Biochem Biophys Res Commun* 2006, **344:**300-307.

56. Park JH, Chang HS, Park CS, Jang AS, Park BL, Rhim TY, Uh ST, Kim YH, Chung IY, Shin HD: **Association analysis of CD40 polymorphisms with asthma and the level of serum total IgE**. *Am J Respir Crit Care Med* 2007, **175:**775-782.

57. Wjst M, Altmuller J, Faus-Kessler T, Braig C, Bahnweg M, Andre E: **Asthma families show transmission disequilibrium of gene variants in the vitamin D metabolism and signalling pathway**. *Respir Res* 2006, **7:**60.

58. Chae SC, Park BL, Park CS, Ryu HJ, Yang YS, Lee SO, Choi YH, Kim EM, Uh ST, Kim YH, Kim KK, Oh B, Chung HT, Kimm K, Shin HD: **Putative association of RUNX1 polymorphisms with IgE levels in a Korean population**. *Exp Mol Med* 2006, **38:**583-588.

59. Kim SH, Oh JM, Kim YS, Palmer LJ, Suh CH, Nahm DH, Park HS: **Cysteinyl leukotriene receptor 1 promoter polymorphism is associated with aspirin-intolerant asthma in males**. *Clin Exp Allergy* 2006, **36:**433-439.

60. Hao L, Sayers I, Cakebread JA, Barton SJ, Beghe B, Holgate ST, Sampson AP, Holloway JW: **The cysteinyl-leukotriene type 1 receptor polymorphism 927T/C is associated with atopy severity but not with asthma**. *Clin Exp Allergy* 2006, **36:**735-741.

61. Arriba-Mendez S, Sanz C, Isidoro-Garcia M, Davild I, Laffond E, Horeno E, Avila C, Lorente F: **927T>C polymorphism of the cysteinyl-leukotriene type-1 receptor (CYSLTR1) gene in children with asthma and atopic dermatitis**. *Pediatr Allergy Immunol* 2006, **17:**323-328.

62. Kim SH, Yang EM, Park HJ, Ye YM, Lee HY, Park HS: **Differential Contribution of the CysLTR1 Gene in Patients with Aspirin Hypersensitivity**. *J Clin Immunol* 2007, **27:**613-619.

63. Thompson MD, Storm van's Gravesande K, Galczenski H, Burnham WM, Siminovitch KA, Zamel N, Slutsky A, Drazen JM, George SR, Evans JF, O'Dowd BF: **A cysteinyl leukotriene 2 receptor variant is associated with atopy in the population of Tristan da Cunha**. *Pharmacogenetics* 2003, **13:**641-649.
