# Supplementary material for: Recent advances in asthma genetics
Source: Respir Res. 2008 Jan 15;9(1):4. doi: 10.1186/1465-9921-9-4 (PMC2244620; doi:10.1186/1465-9921-9-4)
Supplement: Additional file 2 — Studies to detect functional SNPs. The table lists all the genes with functional studies published during 2005–2007 [file 1465-9921-9-4-S2.doc]

| Gene | Chromosomal location | Gene ID | Polymorphism | Experiments | Function | Ref. |
| --- | --- | --- | --- | --- | --- | --- |
| GSTM1 | 1p13.3 | 2944 | Null allele | NA | Absence of the GSTM1 enzyme | [1] |
| FLG | 1q21.3 | 2312 | R510X | Immunohistochemistry | Complete loss of filaggrin peptide production | [2] |
|  |  |  | 2282del4 | Immunohistochemistry | Complete loss of filaggrin peptide production | [2] |
|  |  |  | S2554X and 3321delA | Immunohistologic and ultrastructural observations | Both the variants reduce the keratohyalin granules in the epidermis | [3] |
| TGFB2 | 1q41 | 7042 | -109-->ACAA ins | Luciferase reporter gene assay | Increased the TGF-beta(2) promoter-reporter activity in BEAS2B cells | [4] |
| DPP10 | 2q14.1 | 57628 | WTC122P | EMSA | Allele 2 altered the sequence of promoter element | [5] |
| ICOS | 2q33 | 29851 | -1413G/A | EMSA  ELISA | G allele represented an NF-*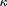*B p50 binding site  A allele increased levels of the Th2 cytokines in PBMCs | [6] |
| CSTA | 3q21 | 1475 | C+344T | Compared decay rate of RNAs transcribed | CSTA +344C mRNA is more than two times less stable than the CSTA +344T mRNA | [7] |
| CD86 | 3q21 | 942 | Ile179Val | Flow cytometry | Val179 induces higher production of both Th1 and Th2 cytokines | [8] |
| DCNP1 | 5q31 | 140947 | c.-1289C>T | Reporter gene assay | C allele affects the gene expression by modulating DNA-protein interaction | [9] |
| CYFIP2 | 5q33.3 | 26999 | CY-In1-8T/C | EMSA | CY-In1-8C deleted the transcription factor binding of GATA | [10] |
|  |  |  | Haplotype of CY-In1-4A/T, CY-In1-8T/C, CY-In1-9G/A, CY-In1-10A/G, IVS3+20G/A, and c.2061C/T | RT-PCR | ATGAGC haplotype increased CYFIP2 expression level in lymphocytes | [10] |
| IL17F | 6p12 | 112744 | rs763780 | Comparison of the recombinant wild-type and mutant IL-17F proteins | H161R variant lacked the ability to activate the mitogen-activated protein kinase pathway, cytokine production, and chemokine production in bronchial epithelial cells | [11] |
| CRTH2 | 11q12-q13.3 | 11251 | Haplotypes of G1544C and G1651A | Transcriptional pulsing experiments | 1544G-1651G haplotype had higher level of reporter mRNA stability | [12] |
| CYSLTR2 | 13q14.12-q21.1 | 57105 | IVS2-37A/G | Luciferase reporter gene assay | G allele has had higher transcriptional activity | [13] |
|  |  |  | 601A>G | Calcium mobilization assay | G allele decreased the potency of LTD_4_ | [14] |
| ECP | 14q24-q31 | 6037 | -393C/T | Luciferase reporter gene assay,  EMSA | -393T allele had lower promoter activity  -393T allele eliminated this C/EBP binding site | [15] |
| PTGDR | 14q22.1 | 5729 | Haplotype of T-549C, C-441T, T–197C | Luciferase reporter gene assay  EMSA | The TCT haplotype had lower reporter activity, and CCC haplotype had higher reporter activity  C allele of T-549C bound GATA1, GATA2 and GATA3; C allele of C-441T eliminated C/EBP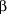 binding site; T allele of T–197C bound an additional DNA-binding protein | [16] |
| EP2 | 14q22 | 5732 | uS5 | Luciferase reporter gene assay  EMSA | A allele showed greater suppressive effects on reporter gene transcription  A allele had higher binding intensity than G allele | [17] |
| IL16 | 15q26.3 | 3603 | −295 T/C | Luciferase reporter gene assay | C allele had higher promoter activity | [18] |
| SOCS1 | 16p13.13 | 8651 | 1478CA>del | Luciferase reporter gene assay  Protein expression and phosphor-STAT1 in nasal fibroblasts | -1478del enhanced the transcriptional level of SOCS1  -1478del induced higher levels of protein expression of SOCS1 and lower phosphorylation of STAT1 stimulated with IFN-beta | [19] |
| TBX21 | 17q21.32 | 30009 | -1993T/C | Luciferase reporter gene assay  EMSA | -1993C allele had higher promoter activity  -1993C allele increased the affinity for an unknown nuclear protein | [20] |
| MMP9 | 20q11.2-q13.1 | 4318 | −1590C>T | Luciferase reporter gene assay | −1590T allele had higher promoter activity | [21] |
| CD40 | 20q12-q13.2 | 958 | -1C>T | EGFP Assay | T allele affected the efficiency of CD40 translation and led to differential expression of CD40 protein | [22] |
| GSTT1 | 22q11.23 | 2952 | Null allele | NA | Absence of the GSTT1 enzyme | [1] |
| CYSLTR1 | Xq13.2-21.1 | 10800 | Haplotype of -634C>T, -475A>C, -336A>G | Luciferase reporter gene assay | TCG haplotype had higher promoter activity | [23] |
|  |  |  | G300S | fluorescence calcium-imaging functional assay | 300S receptor interacts with LTD4 with significantly greater potency | [24] |

**STable2.**

GSTM1: glutathione S-transferase M1; FLG: filaggrin; TGFB2: transforming growth factor, beta 2; DPP10: dipeptidyl-peptidase 10; ICOS: inducible T-cell co-stimulator; CSTA: cystatin A; CD86: CD86 molecule; DCNP1: chromosome 5 open reading frame 20; CYFIP2: cytoplasmic FMR1 interacting protein 2; IL17F: interleukin 17F; CRTH2: chemoattractant receptor-homologous molecule expressed on TH2 cells; CYSLTR2: cysteinyl leukotriene receptor 2; ECP: eosinophil cationic protein; PTGDR: prostaglandin D2 receptor (DP); IL16: interleukin 16; EP2: prostaglandin E receptor 2; SOCS1: suppressor of cytokine signaling 1; TBX21: T-box 21; MMP9: matrix metalloproteinase 9; CD40: CD40 molecule; GSTT1: glutathione S-transferase theta 1; CYSLTR1: cysteinyl leukotriene receptor 1

* SNP name used in the table is from the original paper

EMSA: electrophoretic mobility shift assay; ELISA: Enzyme-Linked ImmunoSorbent Assay; PBMCs: peripheral blood mononuclear cells; RT-PCR: reverse transcriptase PCR;

NA: not available

1. Brasch-Andersen C, Christiansen L, Tan Q, Haagerup A, Vestbo J, Kruse TA: **Possible gene dosage effect of glutathione-S-transferases on atopic asthma: using real-time PCR for quantification of GSTM1 and GSTT1 gene copy numbers**. *Hum Mutat* 2004, **24:**208-214.

2. Smith FJ, Irvine AD, Terron-Kwiatkowski A, Sandilands A, Campbell LE, Zhao Y, Liao H, Evans AT, Goudie DR, Lewis-Jones S, Arseculeratne G, Munro CS, Sergeant A, O'Regan G, Bale SJ, Compton JG, DiGiovanna JJ, Presland RB, Fleckman P, McLean WH: **Loss-of-function mutations in the gene encoding filaggrin cause ichthyosis vulgaris**. *Nat Genet* 2006, **38:**337-342.

3. Nomura T, Sandilands A, Akiyama M, Liao H, Evans AT, Sakai K, Ota M, Sugiura H, Yamamoto K, Sato H, Palmer CN, Smith FJ, McLean WH, Shimizu H: **Unique mutations in the filaggrin gene in Japanese patients with ichthyosis vulgaris and atopic dermatitis**. *J Allergy Clin Immunol* 2007, **119:**434-440.

4. Hatsushika K, Hirota T, Harada M, Sakashita M, Kanzaki M, Takano S, Doi S, Fujita K, Enomoto T, Ebisawa M, Yoshihara S, Sagara H, Fukuda T, Masuyama K, Katoh R, Matsumoto K, Saito H, Ogawa H, Tamari M, Nakao A: **Transforming growth factor-beta(2) polymorphisms are associated with childhood atopic asthma**. *Clin Exp Allergy* 2007, **37:**1165-1174.

5. Allen M, Heinzmann A, Noguchi E, Abecasis G, Broxholme J, Ponting CP, Bhattacharyya S, Tinsley J, Zhang Y, Holt R, Jones EY, Lench N, Carey A, Jones H, Dickens NJ, Dimon C, Nicholls R, Baker C, Xue L, Townsend E, Kabesch M, Weiland SK, Carr D, von Mutius E, Adcock IM, Barnes PJ, Lathrop GM, Edwards M, Moffatt MF, Cookson WO: **Positional cloning of a novel gene influencing asthma from chromosome 2q14**. *Nat Genet* 2003, **35:**258-263.

6. Shilling RA, Pinto JM, Decker DC, Schneider DH, Bandukwala HS, Schneider JR, Camoretti-Mercado B, Ober C, Sperling AI: **Cutting edge: Polymorphisms in the ICOS promoter region are associated with allergic sensitization and Th2 cytokine production**. *J Immunol* 2005, **175:**2061-2065.

7. Vasilopoulos Y, Cork MJ, Teare D, Marinou I, Ward SJ, Duff GW, Tazi-Ahnini R: **A nonsynonymous substitution of cystatin A, a cysteine protease inhibitor of house dust mite protease, leads to decreased mRNA stability and shows a significant association with atopic dermatitis**. *Allergy* 2007, **62:**514-519.

8. Corydon TJ, Haagerup A, Jensen TG, Binderup HG, Petersen MS, Kaltoft K, Vestbo J, Kruse TA, Borglum AD: **A functional CD86 polymorphism associated with asthma and related allergic disorders**. *J Med Genet* 2007, **44:**509-515.

9. Kim Y, Park CS, Shin HD, Choi JW, Cheong HS, Park BL, Choi YH, Jang AS, Park SW, Lee YM, Lee EJ, Park SG, Lee JY, Lee JK, Han BG, Oh B, Kimm K: **A promoter nucleotide variant of the dendritic cell-specific DCNP1 associates with serum IgE levels specific for dust mite allergens among the Korean asthmatics**. *Genes Immun* 2007, **8:**369-378.

10. Noguchi E, Yokouchi Y, Zhang J, Shibuya K, Shibuya A, Bannai M, Tokunaga K, Doi H, Tamari M, Shimizu M, Shirakawa T, Shibasaki M, Ichikawa K, Arinami T: **Positional identification of an asthma susceptibility gene on human chromosome 5q33**. *Am J Respir Crit Care Med* 2005, **172:**183-188.

11. Kawaguchi M, Takahashi D, Hizawa N, Suzuki S, Matsukura S, Kokubu F, Maeda Y, Fukui Y, Konno S, Huang SK, Nishimura M, Adachi M: **IL-17F sequence variant (His161Arg) is associated with protection against asthma and antagonizes wild-type IL-17F activity**. *J Allergy Clin Immunol* 2006, **117:**795-801.

12. Huang JL, Gao PS, Mathias RA, Yao TC, Chen LC, Kuo ML, Hsu SC, Plunkett B, Togias A, Barnes KC, Stellato C, Beaty TH, Huang SK: **Sequence variants of the gene encoding chemoattractant receptor expressed on Th2 cells (CRTH2) are associated with asthma and differentially influence mRNA stability**. *Hum Mol Genet* 2004, **13:**2691-2697.

13. Fukai H, Ogasawara Y, Migita O, Koga M, Ichikawa K, Shibasaki M, Arinami T, Noguchi E: **Association between a polymorphism in cysteinyl leukotriene receptor 2 on chromosome 13q14 and atopic asthma**. *Pharmacogenetics* 2004, **14:**683-690.

14. Pillai SG, Cousens DJ, Barnes AA, Buckley PT, Chiano MN, Hosking LK, Cameron LA, Fling ME, Foley JJ, Green A, Sarau HM, Schmidt DB, Sprankle CS, Blumenthal MN, Vestbo J, Kennedy-Wilson K, Wixted WE, Wagner MJ, Anderson WH, Ignar DM: **A coding polymorphism in the CYSLT2 receptor with reduced affinity to LTD4 is associated with asthma**. *Pharmacogenetics* 2004, **14:**627-633.

15. Noguchi E, Iwama A, Takeda K, Takeda T, Kamioka M, Ichikawa K, Akiba T, Arinami T, Shibasaki M: **The promoter polymorphism in the eosinophil cationic protein gene and its influence on the serum eosinophil cationic protein level**. *Am J Respir Crit Care Med* 2003, **167:**180-184.

16. Oguma T, Palmer LJ, Birben E, Sonna LA, Asano K, Lilly CM: **Role of prostanoid DP receptor variants in susceptibility to asthma**. *N Engl J Med* 2004, **351:**1752-1763.

17. Jinnai N, Sakagami T, Sekigawa T, Kakihara M, Nakajima T, Yoshida K, Goto S, Hasegawa T, Koshino T, Hasegawa Y, Inoue H, Suzuki N, Sano Y, Inoue I: **Polymorphisms in the prostaglandin E2 receptor subtype 2 gene confer susceptibility to aspirin-intolerant asthma: a candidate gene approach**. *Hum Mol Genet* 2004, **13:**3203-3217.

18. Burkart KM, Barton SJ, Holloway JW, Yang IA, Cakebread JA, Cruikshank W, Little F, Jin X, Farrer LA, Clough JB, Keith TP, Holgate S, Center DM, O'Connor GT: **Association of asthma with a functional promoter polymorphism in the IL16 gene**. *J Allergy Clin Immunol* 2006, **117:**86-91.

19. Harada M, Nakashima K, Hirota T, Shimizu M, Doi S, Fujita K, Shirakawa T, Enomoto T, Yoshikawa M, Moriyama H, Matsumoto K, Saito H, Suzuki Y, Nakamura Y, Tamari M: **Functional polymorphism in the suppressor of cytokine signaling 1 gene associated with adult asthma**. *Am J Respir Cell Mol Biol* 2007, **36:**491-496.

20. Akahoshi M, Obara K, Hirota T, Matsuda A, Hasegawa K, Takahashi N, Shimizu M, Nakashima K, Cheng L, Doi S, Fujiwara H, Miyatake A, Fujita K, Higashi N, Taniguchi M, Enomoto T, Mao XQ, Nakashima H, Adra CN, Nakamura Y, Tamari M, Shirakawa T: **Functional promoter polymorphism in the TBX21 gene associated with aspirin-induced asthma**. *Hum Genet* 2005, **117:**16-26.

21. Nakashima K, Hirota T, Obara K, Shimizu M, Doi S, Fujita K, Shirakawa T, Enomoto T, Yoshihara S, Ebisawa M, Matsumoto K, Saito H, Suzuki Y, Nakamura Y, Tamari M: **A functional polymorphism in MMP-9 is associated with childhood atopic asthma**. *Biochem Biophys Res Commun* 2006, **344:**300-307.

22. Park JH, Chang HS, Park CS, Jang AS, Park BL, Rhim TY, Uh ST, Kim YH, Chung IY, Shin HD: **Association analysis of CD40 polymorphisms with asthma and the level of serum total IgE**. *Am J Respir Crit Care Med* 2007, **175:**775-782.

23. Kim SH, Oh JM, Kim YS, Palmer LJ, Suh CH, Nahm DH, Park HS: **Cysteinyl leukotriene receptor 1 promoter polymorphism is associated with aspirin-intolerant asthma in males**. *Clin Exp Allergy* 2006, **36:**433-439.

24. Thompson MD, Capra V, Takasaki J, Maresca G, Rovati GE, Slutsky AS, Lilly C, Zamel N, McIntyre Burnham W, Cole DE, Siminovitch KA: **A functional G300S variant of the cysteinyl leukotriene 1 receptor is associated with atopy in a Tristan da Cunha isolate**. *Pharmacogenet Genomics* 2007, **17:**539-549.
